# Supplementary material for: Bacterial metagenome in plaque, saliva, and tumor samples from individuals with and without OSCC by next-generation sequencing
Source: GigaByte. 2026 Oct 5;2026:gigabyte189. doi: 10.46471/gigabyte.189 (PMC13683955; doi:10.46471/gigabyte.189)
Supplement: Peer review history

# Reviewer report: Review MS


Reviewer name and names of any other individual's who aided in reviewer Morteza banakarDo you understand and agree to our policy of having open and named reviews, and having your review included with the published papers. (If no, please inform the editor that you cannot review this manuscript.)YesIs the language of sufficient quality?YesPlease add additional comments on language quality to clarify if needed
Are all data available and do they match the descriptions in the paper? YesAdditional CommentsAre the data and metadata consistent with relevant minimum information or reporting standards? See GigaDB checklists for examples <a href="http://gigadb.org/site/guide" target="_blank">http://gigadb.org/site/guide</a>YesAdditional CommentsIs the data acquisition clear, complete and methodologically sound?YesAdditional CommentsIs there sufficient detail in the methods and data-processing steps to allow reproduction?YesAdditional CommentsIs there sufficient data validation and statistical analyses of data quality? NoAdditional CommentsIs the validation suitable for this type of data?YesAdditional CommentsIs there sufficient information for others to reuse this dataset or integrate it with other data?YesAdditional CommentsAny Additional Overall Comments to the AuthorThis manuscript presents metagenomic sequencing data from oral samples of OSCC patients and controls. While the dataset has potential reuse value, the manuscript suffers from substantial shortcomings in scientific rigor, methodological clarity, statistical analysis, and data interpretation that must be addressed before publication. --- Major Comments Abstract (Page 2) Line-by-line issues: The abstract lacks specific quantitative results. The statement "Our data revealed profiles of bacterial species" (Page 2) is vague and does not communicate meaningful findings. The authors should report specific taxa that differed significantly between groups, with effect sizes or abundance values. Line 5-7 (Page 2): "The study identified distinct bacterial profiles..." – This describes what was done, not what was found. Revise to state specific differences. Introduction and Background (Page 2-3) Page 2, lines 15-20: The background section does not establish a clear knowledge gap. While the authors note that most studies use 16S rRNA sequencing, they do not explain why metagenomic sequencing would provide novel biological insights beyond taxonomic resolution. Required citation insertion: The authors should explain Oral metagenomics changes in carcinogenesis in the introduction when discussing the advantages of metagenomic approaches over amplicon sequencing for understanding microbial contributions to carcinogenesis (https://doi.org/10.1016/B978-0-443-13531-6.00007-0 Methods (Pages 4-6) Sample Collection (Page 4): Lines 10-15 (Page 4): The inclusion criteria state "history of smoking or alcohol consumption" as required for cancer patients, but Table 2 shows only 1 of 10 OSCC patients reported smoking and 1 reported alcohol intake. This discrepancy suggests either data collection problems or inconsistent application of inclusion criteria. Please clarify. Lines 12-15 (Page 4): "The sample size was determined based on convenience" – This is acceptable for a descriptive/pilot study, but the authors should explicitly state that the study is underpowered for detecting associations and that results are exploratory. Page 5, lines 5-10: The description of plaque collection references teeth 16, 21, 24, 36, 41, and 44. However, OSCC patients with tumors on the tongue, cheek, or lips (Table 2) may have had compromised dentition. Please report how many patients had all six index teeth available and how missing teeth were handled beyond the brief mention. DNA extraction and sequencing (Page 5): Line 15 (Page 5): "DNA was extracted from the samples using a standard protocol" – This is insufficient. Provide the specific kit or method, including lysis conditions (critical for Gram-positive bacteria), enzyme treatments, and elution volumes. Line 20 (Page 5): The Illumina Nextera XT kit was used. Please report fragment sizes, PCR cycle numbers, and whether blank controls or mock communities were included. Bioinformatic Analysis (Page 5): Lines 22-27 (Page 5): Quality trimming parameters are described, but the reference to "Trimmomatic [21]" appears – however, reference 21 is listed on Page 16 as Bolger et al. 2014, but the citation number in the reference list appears inconsistent. Please verify all citations. Page 6, lines 1-5: "Kraken Aligner v1.0" with Kraken DB containing "complete bacterial genomes from RefSeq" – Which version of RefSeq? Was any filtering applied for low-quality genomes? Did the authors consider using Bracken for abundance estimation rather than raw Kraken counts? Statistical Analysis (Page 6): Line 10 (Page 6): Normalization to 10,000 reads per sample. Table 1 shows final reads ranging from ~75,000 to ~100,000 per sample, so 10,000 is reasonable. However, please report how many samples were excluded because they did not meet this threshold. Lines 11-13 (Page 6): Mann-Whitney U tests with FDR correction. With only 10 patients per group and hundreds of taxa, this analysis is severely underpowered. The authors should acknowledge that FDR correction with such small sample sizes may produce both false negatives and false positives. Consider presenting unadjusted p-values alongside FDR-adjusted values for transparency. Page 6, lines 14-16: PCoA using Bray-Curtis dissimilarity – Please report whether a PERMANOVA was performed to test for significant differences between groups, and if so, the R² and p-values. Results (Pages 6-12) Table 1 (Page 7): The formatting is severely corrupted. The table appears to show reads before and after trimming, but the columns are misaligned. For example, "trimmomatic-0.32.jar PE-phed33" appears to be a typo (should be "phred33"). Please reformat this table completely. Page 7, Table 2: The control group shows "Yes = 1" for alcohol intake but has no tumor localization (NA). However, the inclusion criteria for controls did not mention excluding drinkers – please clarify whether the control group smoker/drinker were matched to cases. The statement that 90% of participants were women is notable given that OSCC is more common in men globally – please discuss this limitation. Page 7, lines 5-10: The oral hygiene disparity (80% of controls with good hygiene vs. 20% of cases) is a major potential confounder that the authors acknowledge but do not address statistically. Multivariable modeling is not possible with n=10, but the authors should be more cautious in attributing microbial differences to OSCC rather than oral hygiene. Page 7, lines 20-25 (GC content): The statement that "there is no evidence of extreme outliers or secondary peaks that would clearly suggest contamination" is reassuring, but did the authors screen for reads mapping to human genome? Contamination by human DNA is common in tumor samples. Page 8, Figure 1: The figure is referenced but not visible in the provided text. Please ensure all figures are properly formatted and legible. Page 8, lines 15-20 (Species overlap analysis): The authors report 164 bacterial species in OSCC patients and 139 in controls. How were species defined? Did the authors use species-level classification from Kraken, which is known to have variable accuracy? This should be addressed in limitations. Page 10, lines 5-15: The statement that "Escherichia coli, Lacrimispora sp. HJ-01, and Brevibacterium casei" showed increased abundance in tumors is interesting but lacks statistical support. Please report which comparisons were significant after FDR correction. Required citation insertion: The authors should cite Differences in the Oral Microbiome Between Patients With and Without Oral Squamous Cell Carcinoma (https://doi.org/10.1111/jop.70099) in the Results or Discussion section when comparing their findings on taxa associated with OSCC to previously reported differences. Page 11, Figure 4: The "unknown barcode" sample is identified as an outlier with ~193 species. Please identify what this sample was (which patient, which tissue type) and justify its inclusion. If it represents a technical artifact or contamination, consider excluding it. Page 12, lines 5-10 (PCoA description): The description states that "Patient Tumor samples (MT) consistently cluster in the upper part of the plot" and "Case Plaque (CP) and Patient Plaque (MP) samples tend to occupy the left side." Without statistical testing (PERMANOVA), these visual interpretations are subjective. Discussion (Pages 12-13) Page 12, lines 15-25: The discussion cites many established concepts about dysbiosis and inflammation but does not adequately contextualize the novel findings of this study. Which specific bacterial associations with OSCC have not been previously reported? Which confirm previous work? Page 13, lines 1-5: The authors mention "Prevotella multiformis and Simonciella munnelli" as present in OSCC plaque/saliva, and "Streptococcus cristatus" in controls. However, these are not mentioned in the Results section. Please ensure consistency between Results and Discussion. Page 13, lines 5-8: "Streptococcus periodonticum, Pajuensenia hongkongensis, and Leptotrichia buccalis" are noted as highly represented in OSCC. Were these statistically significant after multiple testing correction? Limitations (Page 13, lines 10-18): The authors list several confounding factors but do not mention the small sample size as a primary limitation. Please add explicit acknowledgment that n=10 per group severely limits generalizability and statistical power. Re-use potential (Page 13): This section is appropriate for a data paper but should be condensed. The statement that "All analyses reported here can be reproduced using the programs and databases referenced herein" requires that all versions are specified – please add software versions for Kraken, Trimmomatic, R packages, and the exact database version numbers. References (Pages 15-18) The reference list has formatting inconsistencies. Several references lack DOIs or have incomplete information (e.g., reference 29 on Page 17 is incomplete). Reference 21 is cited in text but appears as "[21]" on Page 16 – the numbering seems inconsistent with earlier citations. The authors should verify all citations against the main text, as there appear to be discrepancies between in-text citation numbers and the reference list. --- Minor Comments 1. Page 2, title: The title includes "# (GIGA)byte PAPER" – this appears to be journal formatting and should be removed from the manuscript file. 2. Page 2, author affiliations: The dagger symbols (†) for corresponding authors are not explained – please indicate what these denote. 3. Page 5, line 23: "Avaiable" should be "Available" (typo in FastQC URL description). 4. Page 6, line 11: "rarefaction to a uniform sequencing depth of 10,000 reads" – Please note that rarefaction discards data and is controversial; alternative approaches like CSS normalization or including library size as a covariate could be mentioned. 5. Page 7, Table 1: "Length pb" should be "Length bp" (base pairs). The read lengths show ranges (100-266 and 100-242) – these should be reported as mean ± SD or median with IQR. 6. Page 7, Table 2: The formatting suggests "Good = 8 Regular = 2 Bad = 0" for control oral health – please clarify the scoring system used. 7. Page 10, line 3: "Cardioacterium hominis" appears to be a typo – should this be "Cardiobacterium hominis"? 8. Page 10, line 4: "Treponema sp. Marseille-Q4132" – please italicize species names throughout. 9. Page 13, Data Availability: The BioProject ID is listed as "PRINA1165312" – this appears to be a typo; BioProject accessions typically start with PRJNA (e.g., PRJNA1165312). Please verify. 10. Page 14, Author Contributions: The use of "Andres Ignacio Chala" in contributions but "Andrés Ignacio Chalar" in author list – please standardize spelling. --- Ethical and Data Concerns · The ethics approval number (OD-0149) and CIEFOPUJ Act 11 of 2015 are provided – this is adequate. · The data availability statement includes a BioProject ID (pending verification), which should be active and accessible upon publication.RecommendationMajor Revision


# Reviewer report: Review MS


Reviewer name and names of any other individual's who aided in reviewer Chen Chen, Wei XieDo you understand and agree to our policy of having open and named reviews, and having your review included with the published papers. (If no, please inform the editor that you cannot review this manuscript.)YesIs the language of sufficient quality?NoPlease add additional comments on language quality to clarify if needed
The manuscript is generally understandable; however, several sections would benefit from language editing to improve clarity, consistency, and readability. In particular, some methodological descriptions are lengthy and could be presented more clearly. Professional language editing is recommended.Are all data available and do they match the descriptions in the paper? NoAdditional CommentsThe paper reports 49 metagenomic samples analyzed, but the NCBI BioProject (PRJNA1165312) contains only 46 samples. The authors should clarify this discrepancy by providing details on which samples were excluded during data processing and the specific reasons for their exclusion.Are the data and metadata consistent with relevant minimum information or reporting standards? See GigaDB checklists for examples <a href="http://gigadb.org/site/guide" target="_blank">http://gigadb.org/site/guide</a>NoAdditional CommentsThe study includes 10 OSCC patients and 10 healthy controls. While the dataset is valuable, the relatively small sample size may limit statistical power and the generalizability of downstream analyses. This limitation should be more clearly acknowledged and discussed in the manuscript.Is the data acquisition clear, complete and methodologically sound?NoAdditional CommentsWhile the sample collection and metagenomic sequencing procedures are clearly described, the limited sample size and relatively shallow sequencing depth, particularly for tumor samples, may hinder the robust detection of low-abundance taxa. Additionally, the notable disparity in oral hygiene between the two groups was not strictly matched, which could potentially introduce confounding biases.Is there sufficient detail in the methods and data-processing steps to allow reproduction?NoAdditional CommentsSome methodological details could be described more clearly to improve reproducibility, including parameter settings for Trimmomatic, host read removal procedures, software versions, and other analytical parameters. In addition, the approach of rarefying all samples to 10,000 reads for diversity analysis would benefit from a brief justification. For the beta diversity analysis, it may be helpful to include statistical testing (e.g., PERMANOVA) in addition to the PCoA visualization to support group comparisons.Is there sufficient data validation and statistical analyses of data quality? YesAdditional CommentsWhile the authors adequately demonstrate basic raw sequencing quality via FastQC, this is insufficient for comprehensive metagenomic data validation. Critical statistical quality controls—such as host DNA contamination removal efficiency and rarefaction analysis for sequencing depth saturation—are missing. Furthermore, the unexplained discrepancy between 49 and 46 samples, prevents independent verification of the data quality and statistical conclusions.Is the validation suitable for this type of data?NoAdditional CommentsFastQC is applicable as a raw data quality assessment tool and serves as the standard starting point for metagenomic quality control workflows. However, a FastQC report alone is insufficient to constitute comprehensive metagenomic data validation.Is there sufficient information for others to reuse this dataset or integrate it with other data?YesAdditional CommentsThe deposited data indicates the sampling sites, providing a basic foundation for reuse. However, the authors still need to provide the case and control grouping information, along with other relevant clinical metadata. Until these critical details are supplemented, meaningful integration of this dataset with other cohorts will remain difficult.Any Additional Overall Comments to the AuthorAlthough this study holds scientific value, the description of the metagenomic workflow is insufficient, lacking key QC metrics such as host contamination removal rates and rarefaction curves. Furthermore, the authors must clarify the discrepancy between the 49 reported and 46 deposited samples, and provide case/control grouping metadata to ensure data reuse. Finally, the manuscript requires language polishing.RecommendationMajor Revision


# Reviewer report: Re-Review MS


Indicate in the comments box below whether you are happy with the changes made or if the manuscript is unacceptable.Comments on revised manuscriptThe authors sufficiently replied to all my comments.Indicate in the comments box below whether you are happy with the changes made or if the manuscript is unacceptable.Comments on revised manuscriptThe authors sufficiently replied to all my comments.


# Editor report: Accept


Editor’s AssessmentThe manuscript is ready for formal acceptance.Editor’s AssessmentThe manuscript is ready for formal acceptance.
